# The defective gut colonization of *Candida albicans hog1* MAPK mutants is restored by overexpressing the transcriptional regulator of the white opaque transition *WOR1*

**DOI:** 10.1080/21505594.2023.2174294

**Published:** 2023-02-09

**Authors:** Elvira Román, Daniel Prieto, Susana Hidalgo-Vico, Rebeca Alonso-Monge, Jesús Pla

**Affiliations:** Departamento de Microbiología y Parasitología, Facultad de Farmacia, Universidad Complutense de Madrid, Madrid, Spain

**Keywords:** Candida albicans, gut colonization, commensalism, Wor1, Hog1, MAPK

## Abstract

The transcriptional master regulator of the white opaque transition of *Candida albicans WOR1* is important for the adaptation to the commensal lifestyle in the mammalian gut, a major source of invasive candidiasis. We have generated cells that overproduce Wor1 in mutants defective in the Hog1 MAP kinase, defective in several stress responses and unable to colonize the mice gut. *WOR1* overexpression allows *hog1* to be established as a commensal in the murine gut in a commensalism model and even compete with wild-type *C. albicans* cells for establishment. This increased fitness correlates with an enhanced ability to adhere to biotic surfaces as well as increased proteinase and phospholipase production and a decrease in filamentation in vitro. We also show that *hog1* WOR1^OE^ are avirulent in a systemic candidiasis model in mice.

## Introduction

*C. albicans* is a member of the vaginal and the gastrointestinal human mycobiota. It is estimated that more than 50% of human individuals without an underlying pathology are colonized with this fungus and this value may be higher as colonization depends on the individual conditions. Broad spectrum antibacterial antibiotics, diabetes, and certain immunological disorders (among others) favour *C. albicans* overgrowth and disease [1,2]. Alteration of host defences facilitates the access of *C. albicans* to other noncanonical body locations causing diseases called candidiasis which are frequently life threatening and have high mortalities [3,4]. Although catheters are an important cause of nosocomial infections, most candidiasis have an endogenous origin [5–7] being the fungal pool in the gut a major source of dissemination. Although several virulence factors have been identified in the last years using acute systemic infection murine model, the identification of factors promoting colonization may be crucial to our understanding of *Candida* infections. Therapies directed against these factors and/or processes may eliminate *C. albicans* from the gut; however, they may also be detrimental in certain situations given the underlying benefits of colonizing this niche [8]. Therefore, it is important to precisely define which processes in the fungus and the host regulate commensalism to potentially manipulate this interaction to improve human health.

Different genes have been shown to mediate gut colonization in mice [9–11]. The Efg1 morphogenetic regulator was one of the first genes discovered playing a role in commensalism [12]; *efg1* mutants outcompeted wild-type cells at early time points after gavage in mice and showed increased fungal burdens in the intestine. Efg1 is also a key regulator of the white opaque (**wo**) transition [13], a genetic program that prepares cells for mating and leads to tetraploid cell formation [14]. **wo** switching is inhibited by the **a**1-α2 repressor, only occurs in **a** or α cells and it is environmentally regulated [15], being favoured by relatively low (21ºC) temperatures and high CO_2_ levels. The *WOR1* gene was identified as an **a**1α2 repressed white-phase gene and its deletion blocked opaque formation, while its overexpression induces an *en masse* conversion to opaque cells [16–18]. *WOR1* is involved in the adaptation to the commensal lifestyle by generating GUT (Gastrointestinal indUced Transition) cells following the passage through the mouse gastrointestinal tract [19]. Deletion of *WOR1* results in reduced fitness in the gut but its overexpression (WOR1^OE^) from the strong *TDH3* promoter enhances fitness in this mouse model of commensalism. GUT cells could be differentiated from “standard” opaque cells due to their increased *in vivo* fitness in this niche, by their surface ultrastructural details and different transcriptional programs [19].

The relationship between opaque and GUT cells raises several questions. The white-to-opaque transition at 37ºC is favoured under certain conditions, such as high levels of CO_2_ (equivalent to those found in certain niches and the gut), N-acetylglucosamine (which is produced by certain bacteria in the gut) and the anaerobic environment [20–22], suggesting that the optimal state for gut colonization is the opaque one. Opaque cells colonize skin more readily [23] but they are less virulent than white cells in a mouse model of systemic infection [24]. Furthermore, while opaque cells are more resistant to phagocyte-mediated killing [25–28] they are severely attenuated for commensalism [19]. Overexpression of *WOR1* has a pleiotropic effect on *C. albicans* cells: it modifies the respiratory metabolism, rendering cells more susceptible to electron-chain inhibitors and to bile salts and enhances adhesion to the gastrointestinal mucosa [29]. These changes are sustained by transcriptomic [19] and proteomic analyses [30]. For example, *WOR1*^OE^ cells reduce the expression of the isocitrate lyase encoded by the *ICL1* gene, an enzyme of the glyoxylate cycle, which is consistent with the profound and complete transcriptional adaptation program underlying commensal adaptation [31]. Within the gut, *C. albicans* cells must face diverse and complex signals generated by the host and the commensal microbiota and adapt to nutrients different from those commonly used under laboratory conditions [11,32]. It seems plausible that MAPK signalling pathways could be involved in adaptation to colonization given their role in sensing osmotic, oxidative, and cell wall stress [33–35]. The HOG route has been shown to play an important role in the colonization of the mouse gut, as both *hog1* and *pbs2* (the Hog1 MAPKK) are defective in fitness when competing a wt strain in co-colonization experiments [36] but the mechanisms responsible for this behaviour are not completely understood. We show here that overexpression of *WOR1* restores fitness to the *hog1* mutant in the gastrointestinal tract; furthermore, *hog1* WOR1^OE^ cells increase their adhesion to the gastrointestinal mucosa and do not show the hyperfilamentous phenotype of *hog1* mutants [37], thus supporting the connection between filamentation, adhesion, and gut colonization.

## Results

### WOR1^OE^ suppresses fitness defects of *hog1* mutants

As Wor1 promotes gut colonization [19], we wondered whether overexpression of *WOR1* could suppress the failure of *hog1* mutants to colonize [36]. A strain overexpressing a myc tagged version of *WOR1* was generated in a *hog1* background (see Materials and Methods). This new strain, *hog1*-WOR1^OE^, produced Wor1 in a doxycycline repressible manner (Fig. S1A). The amount of *WOR1* mRNA determined by qPCR revealed a ≈ eightfold higher *WOR1* expression in the *hog1*-WOR1^OE^ compared to the *hog1*-pNRUe empty vector control strain and≈20-fold higher compared to the wild-type pNRUe (Fig. S1B). *hog1-*WOR1^OE^ cells showed phenotypes previously described in the CAI4-WOR1^OE^ wildtype background [29,30], such as larger cells (Fig. S1C) and increased phloxine B staining (Fig. S1), while remaining a/α mating type, and these phenotypes were dependent on the presence of doxycycline.

**Figure 1.**
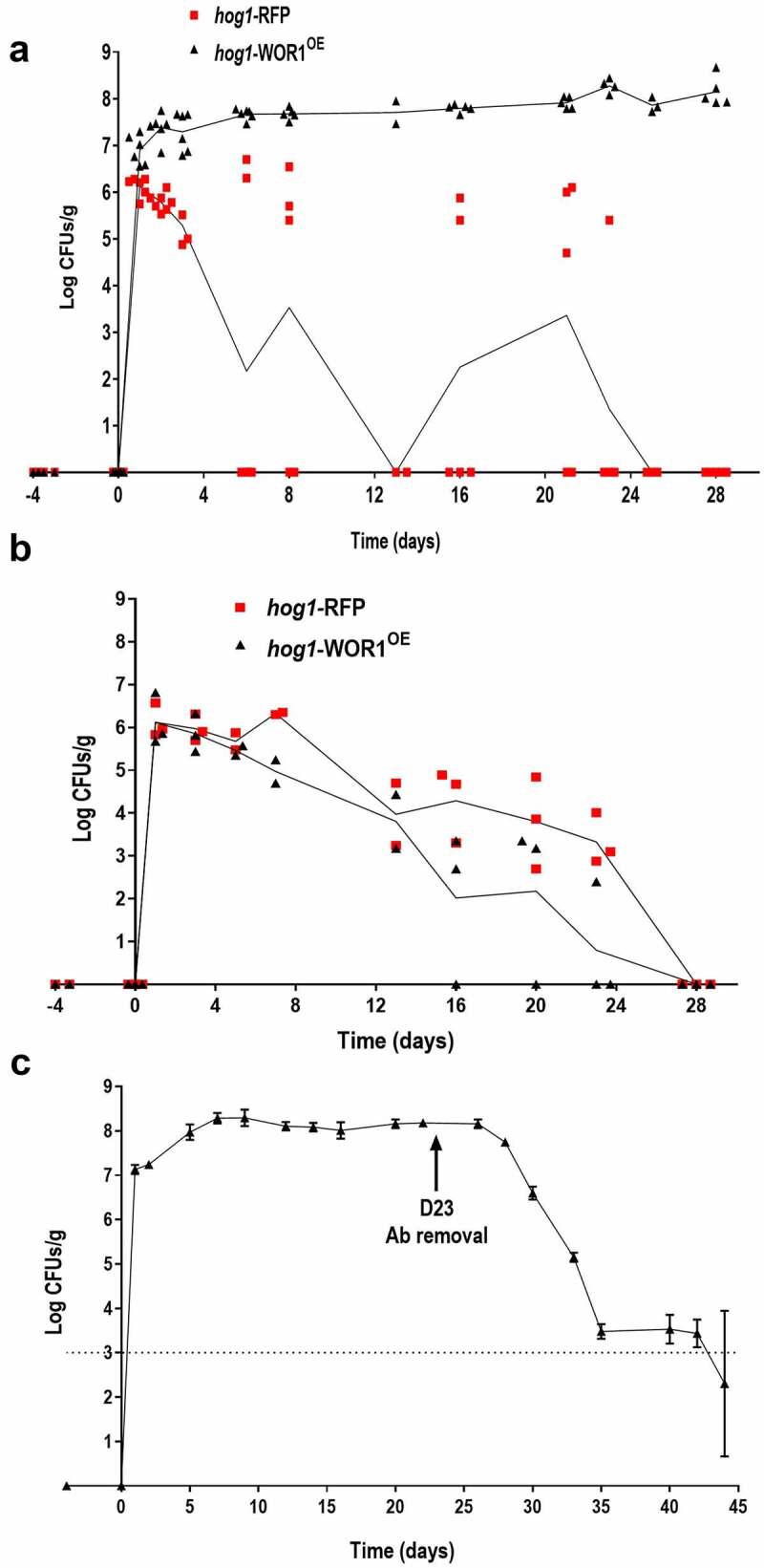
*WOR1* overexpression suppresses fitness defects of *hog1* mutants.

Note: Competition assays of *hog1*-RFP and *hog1*-WOR1^OE^ (HW1) cells in C57BL/6 mice (n = 4–6). Oral antibiotic therapy without (A) or with doxycycline (B) was administered 4 days before a gavage of 10^7^ cells in a 1:1 mixture (day 0) and maintained throughout the experiment. Fungal loads detected by CFUs counting along the time (days) are represented as the mean ± SD and are spotted at the indicated time points (days). C) Colonization loads of the *hog1*-WOR1^OE^ strain in a single colonization assay.

Colonization competitive assays of *hog1*-WOR1^OE^ and *hog1*-RFP strain showed that shortly after gavage (1–2 days), *hog1*-WOR1^OE^ cells achieved high levels of colonization (10^6^-10^7^ CFU/g), while *hog1*-RFP loads were significantly lower (10^6^-10^5^ CFU/g). After this period, *hog1*-RFP fungal colonization was around or below the detection levels (≈10^4^ CFU/g) in ~ 70% of mice and was finally lost in all animals after 4 weeks (Figure 1a). In clear contrast, *hog1*-WOR1^OE^ levels increased up to 10^8^ CFU/g during this period. This later effect was due to *WOR1* overexpression, as it was not observed when its expression was repressed in mice that received aCT (autoclaved chlortetracycline, a compound with a significantly reduced antibiotic activity while maintaining the regulatory effect of doxycycline) in drinking water during the colonization experiment (Figure 1b). In this case, both strains similarly disappeared after 3–4 weeks consistent with the behaviour of *hog1* mutants [36]. To confirm that *WOR1* overexpression enables *hog1* to colonize the mouse gut, we followed *hog1*-WOR1^OE^ loads in colonization assays using only this strain. As shown (Figure 1c), colonization loads at day 1 after gavage were as high as 10^7^ CFU/g, reaching 10^8^ CFU/g at day 7. These loads were maintained until antibiotic treatment was removed from the drinking water at day 23, where they decreased below the limit of detection at day 45.

**Figure 2.**
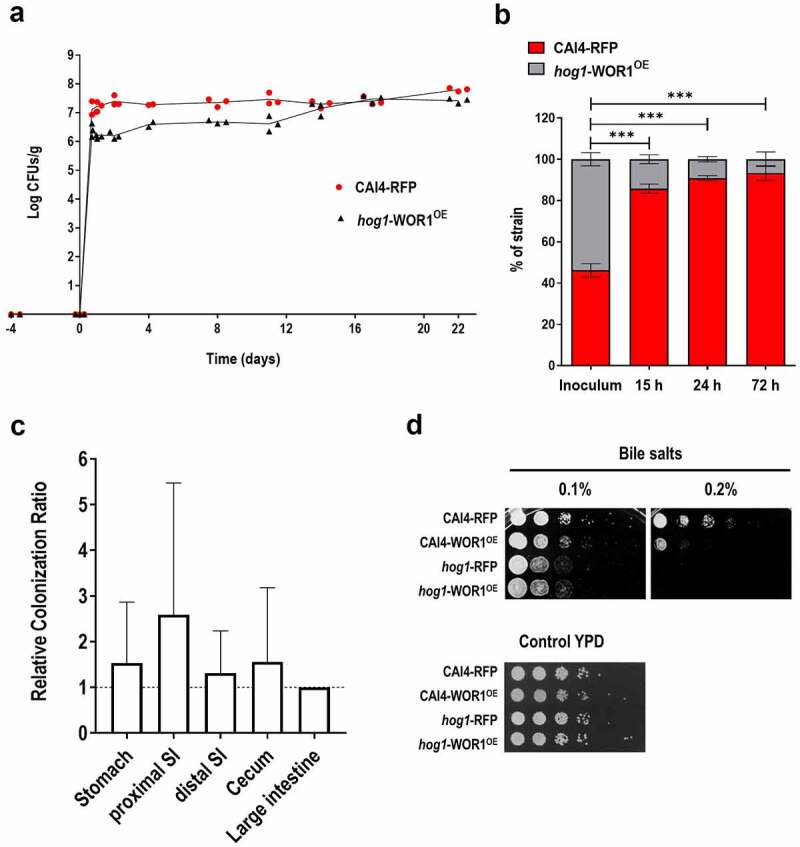
Competition of hog1-WOR1^OE^ cells with wild-type cells.

Note: Antibiotic therapy in drinking water was given to mice (n = 4–6) starting 4 days before a gavage of 10^7^ cells in a 1:1 mixture of *hog1*-WOR1^OE^ (HW1) and CAI4-RFP (day 0) was administered. Fungal loads detected by CFUs counting along the time (days) are represented as the mean ± SD. B) Comparison of percentages of the CAI4-RFP and *hog1*-WOR1^OE^ strains in the inoculum and in fecal samples at early time points (mean± SD). Ordinary one-way ANOVA plus Dunnett multiple comparisons test was used for statistical analyses. *p < 0.05, **p < 0.01, ***p < 0.001, ****p < 0.0001. C) *Postmortem* analysis of the relative colonization ratios of CAI4-RFP/*hog1*-WOR1^OE^ along the gastrointestinal tract at day 25 is represented as the mean± SD. D) Tenfold serial dilutions of overnight growing cultures of the indicated strains were spotted on YPD plates supplemented with 0.1% and 0.2% Bile salts and incubated at 37°C for 48 h before being scanned.

We next addressed whether increased fitness of *hog1*-WOR1^OE^ cells is enough to allow cells to compete against wild-type *HOG1* cells. In a standard competitive colonization assay between hog1-WOR1^OE^ and the parental strain (CAI4-RFP), we observed that *hog1*-WOR1^OE^ colonization load was approximately one log_10_ lower than wt during the first 10–12 days, but eventually recovered and reached similar levels to wt from day 14 onwards after gavage (Figure 2a). This behaviour partially resembles the phenotype of CAI4-WOR1^OE^ under equivalent conditions, where during the initial colonization stages lower colonization rates were observed [29]. In fact, when the relative amounts of each strain were analysed in the first 72 hours, we observed that 15 hours after gavage of equivalent amounts of each strain, CAI4-RFP represented 80% of the total fungal cells recovered and up to 90–95% at 48 and 72 hours (Figure 2b). However, *post-mortem* analyses at day 25 revealed that there were no significant differences in the relative proportion of both strains in the stomach, proximal small intestine, distal small intestine, and caecum, being close to 1 (Figure 2c), indicating that there is no bias towards colonization of a specific region at day 25. As a defective colonization of the initial portions of the gut has been suggested to be mediated by the susceptibility to bile salts [36], we determined the susceptibility of these strains in a standard drop assay on solid media. As shown in Figure 2d, overexpression of *WOR1* did not result in an enhanced sensitivity to bile salts (tested at 0.1% and 0.2%) in a *hog1* background as occurs in wild-type cells. These experiments suggest that overexpression of *WOR1* is enough to suppress the deficient fitness associated with *hog1* cells in this mouse colonization model by mechanisms not involving susceptibility to bile salts.

### WOR1^OE^ enhances adhesion to the mouse gastrointestinal mucosa

**Figure 3.**
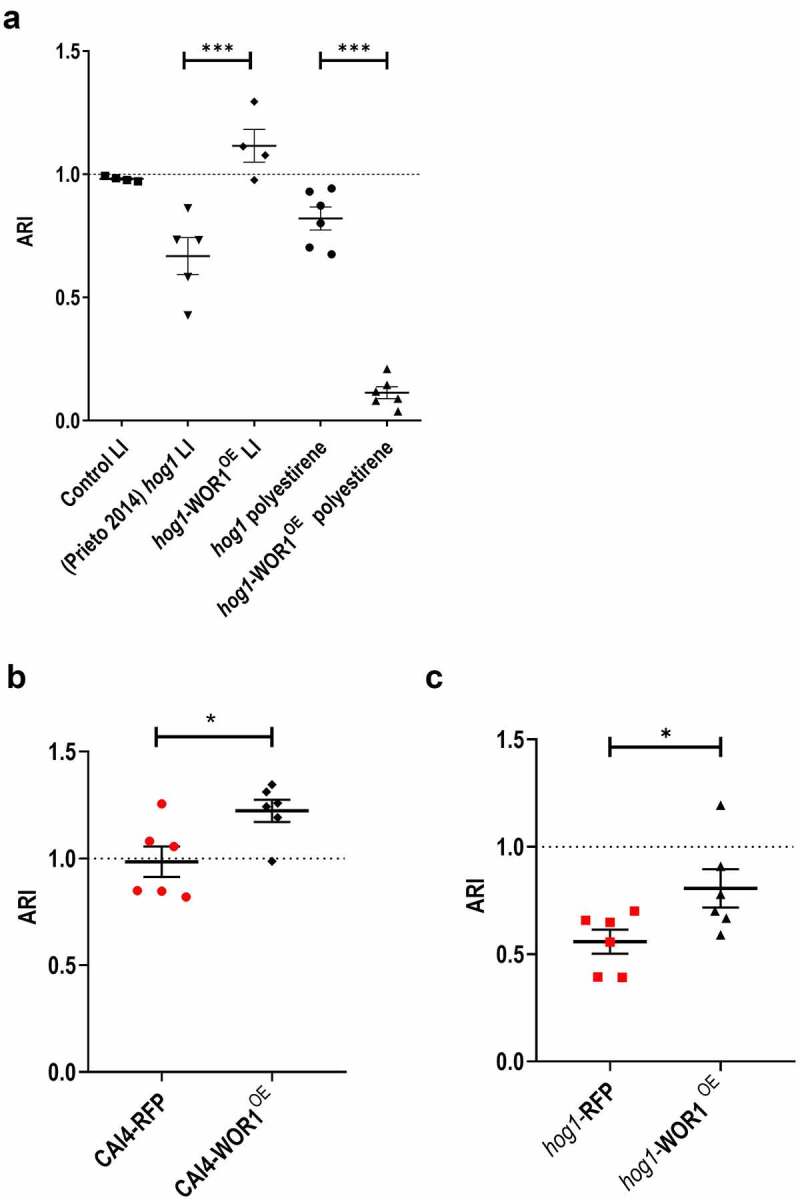
Effect of overexpression of *WOR1* in *hog1* cells adhesion.

Note: The Adhesion Relative Index (ARI) was determined for the CAI4-RFP (control), *hog1,* and *hog1*-WOR1^OE^ strains compared to the wild-type strain CAI4-RFP in the large intestine (LI) and/or polystyrene (A) or to the adenocarcinoma cell-line HT29 (B and C). Individual values are plotted with the mean ± SEM. Ordinary one-way ANOVA plus Tukey multiple comparisons test or unpaired t-test was used for statistical analyses. Ns no significant, *p < 0.05, **p < 0.01, ***p < 0.001.

**Figure 4.**
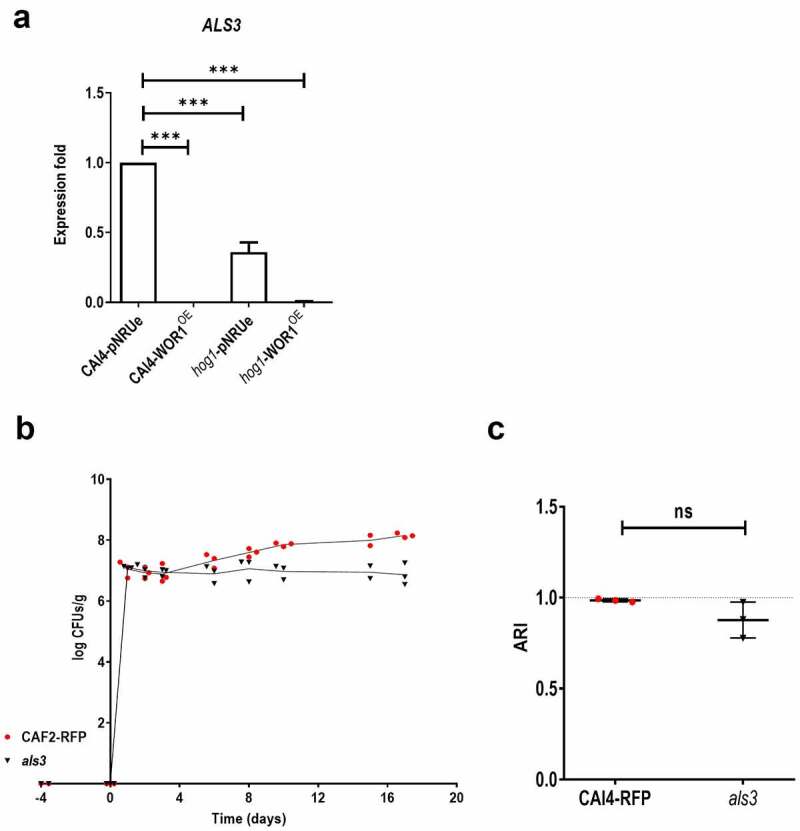
Role of Als3 in fitness.

Note: *ALS3* transcription level was measured by quantitative RT-qPCR.*ACT1* mRNA was used as internal control and fold increase over *ALS3* mRNA in the CAI4-pNRUe in the indicated strains was represented. Each qPCR (three biological replicates) provided three intraexperiment values and are shown as the mean ± SD. Ordinary one-way ANOVA plus Dunnett multiple comparisons test was used for statistical analyses. *p < 0.05, **p < 0.01, ***p < 0.001, ****p < 0.0001. B) Competition between wild-type CAF2-RFP and *als3* mutant strain colonization in C57BL/6 antibiotic treated mice (n=3). Fungal loads determined by CFU counting of each strain are represented at different time points (days) as mean ± SD. C) The Adhesion Relative Index (ARI) was determined for the *als3* mutant compared to the wild-type strain CAF2-RFP in the large intestine *ex vivo* like Figure 3. Paired t-test was performed for statistical analyses. ns, not significant.

Recently, certain *C. albicans* adhesins have been shown to play a role in the adaptation to the mammalian gut due to the induced adaptive immune response [38,39]. As adhesion could play a role in colonization, we first analysed how mutant strains adhered to either abiotic (polystyrene) or biotic (gut mucosa) surfaces by determining the adhesion relative index (see Materials and Methods). The overexpression of *WOR1* resulted in an increased adhesion (ARI = 1.116 ± 0.13) to the large intestine mucosa compared to *hog1* (ARI = 0.67 ± 0.167), an effect very significative given that *hog1* is less adherent than a wt strain [36]. In striking contrast, adhesion to polystyrene followed an opposite pattern and was decreased in *hog1*-WOR1^OE^ (ARI = 0.12 ± 0.06) compared to *hog1* (ARI = 0.82 ± 0.11) (Figure 3a). A similar effect was observed in the adhesion to the human colon adenocarcinoma cell line, HT29, where overexpression of *WOR1* increased the ARI~1.2-fold compared to the isogenic wt strain or ~1.45 × if we compare *hog1*-WOR1^OE^ to *hog1* mutant cells. Similarly, we observed that the *hog1* background adheres less efficiently than the wt strain to this cell line (Figure 3c). We quantified the expression levels of different adhesins under standard laboratory conditions, such as the agglutinin-like sequence genes *ALS3* and *ALS6*, as well as the GPI-anchored cell wall adhesin *EAP1*. *ALS3* expression was found to be ~ 2.8-fold lower in *hog1* (0.36 ± 0.069) compared to a wt strain that was completely repressed in both backgrounds when overexpressing *WOR1* (Figure 4a). No significant differences were found for either *ALS6* or *EAP1* in WOR1^OE^ cells (Fig. S2). We next compared the level of colonization in a competitive assay of an *als3* mutant and a wt strain expressing RFP. Both strains behaved similar in the first week although *als3* colonization was 10-fold lower at day 10 after gavage (Figure 4b). No significant differences in an *ex vivo* adhesion to mouse gastrointestinal mucosa were found between wt (0.985 ± 0.009) and the *als3* mutant (0.877 ± 0.099) (Figure 4c). These data indicate that *WOR1* overexpression results in changes in adhesion to gut mucosa and influences the expression of *ALS3 in vitro*, but this is not the only mechanism responsible for the increased fitness of *WOR1*^*OE*^ cells.

### Overproduction of Wor1 suppresses *hog1* filamentation

**Figure 5.**
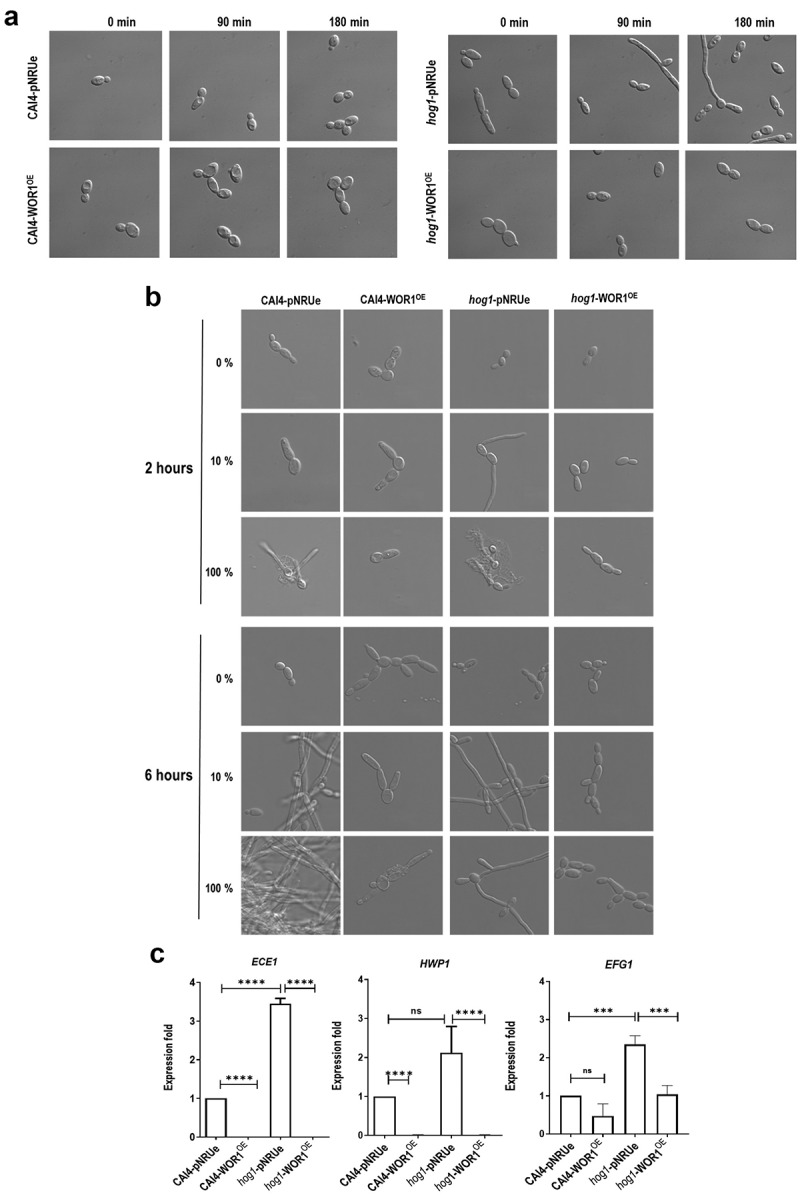
Effect of Wor1 overproduction in *hog1* morphogenesis.

Note: A) Overnight cultures of the indicated strains were diluted in fresh YPD liquid media and incubate at 37ºC. At 0, 90, and 180 min after dilution, samples were obtained from cultures and photographed. B) Overnight cultures of the indicated strains were diluted in fresh YPD, YPD 10% FBS, or FBS 100% and incubated at 37ºC. Representative microscopic images at 2 and 6 hours are shown. C) Relative transcript levels of *ECE1*, *HWP1,* and *EFG1* in the indicated strains are compared to the CAI4-pNRUe control strains determined by RT-qPCR using *ACT1* transcript as internal control. Each qPCR (three biological replicates) provided three intraexperiment values and are shown as the mean ± SD. Ordinary one-way ANOVA plus Tukey multiple comparisons test was used for statistical analyses. *p < 0.05, **p < 0.01, ***p < 0.001, ****p < 0.0001. ns, not significant.

The ability to switch between yeast and hyphae is related to pathogenesis and has been linked to the commensal state as long-termed GI colonization by *C. albicans* select hyper-fitted fungal cells that show filamentation defects [40]. We hypothesized that the ability of *hog1*-WOR1^OE^ cells to colonize could involve the suppression of the hyperfilamentous phenotype of *hog1* mutants [37]. We then studied the behaviour of overnight growing cells after dilution in fresh YPD and followed filamentous growth at different time points. Overexpression of *WOR1* completely abolished *hog1* filamentation (easily visible at short time points, 90 and 180 min), while no filaments were observed either in wt or in CAI4-WOR1^OE^ (Figure 5a). A similar result was observed when cells were exposed to low serum concentrations: while *hog1* cells display a clear filamentous phenotype in sub (10% serum) and fully (100% serum) inducing conditions, the frequency of germination and/or the extent of the filament size was much reduced in *hog1*-WOR1^OE^ cells at 2, 6 (Figure 5b) and 24 (Fig. S3) hours at 37ºC. We also quantified the expression of some hyphae-specific genes like *ECE1* and *HWP1* and the transcription factor *EFG1*, responsible for maintaining the white-phase cell type (Figure 5c). All of them were shown to be upregulated more than twofold in the *hog1* mutant (*hog1*-pNRUe) compared to the CAI4-pNRUe (*ECE1*: 3.447 ± 0.24; *HWP1*: 2.123 ± 1.17; *EFG1*: 2.35 ± 0.23). However, when *WOR1* was overexpressed, *ECE1* and *HWP1* expression was completely blocked, while *EFG1* decreased to wt values (1.037 ± 0.24). These results demonstrate that overproduction of Wor1 is sufficient to suppress the hyperfilamentous phenotype of *HOG1* deletion and the expression of filamentation associated genes.

### Phospholipase and protease activities increases upon overexpression of *WOR1*

**Figure 6.**
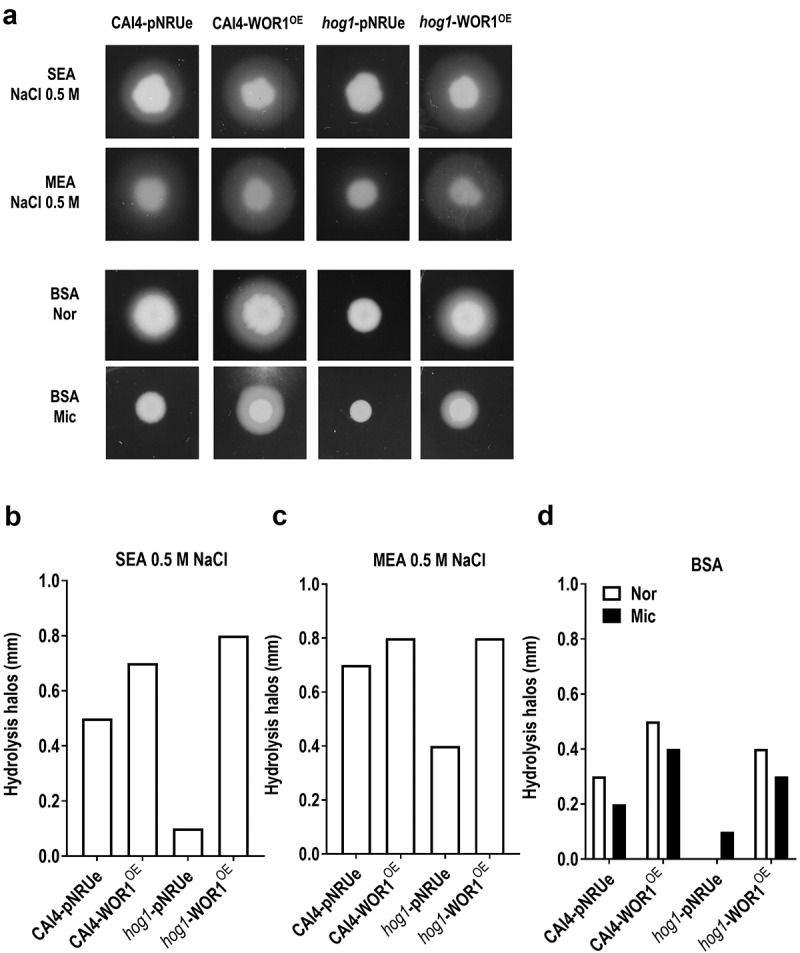
Determination of phospholipase and protease activity in WOR1^OE^ cells.

Note: Phospholipase and protease activities were determined on MEA and SEA 0.5 M NaCl or BSA agar plates, respectively, from overnight growing cells. 6.25x10^4^ cells were deposited onto agar plates and were incubated 96 (MEA and SEA plates in normoxia) or 120 hours (BSA plates in normoxia, Nor, or microaerophilia, Mic) before scanned (A). Hydrolysis halos (mm) were measured and represented for all the strains on SEA (B), MEA (C) and BSA (D) plates.

Phospholipases and proteases have been related to invasion, damage, and pathogenesis in *C. albicans* [41]. We tried to determine whether *WOR1* could play a role in regulating these activities *in vitro*. Phospholipase activity was measured in SEA (Sabouraud egg agar) and MEA (malt egg agar) in the presence of NaCl, and hydrolysis halos were measured after 96 hours of incubation in normoxia. We used 0.5 M NaCl since *hog1* mutants show growth defects at higher concentrations. No effect on growth upon osmotic stress was observed in cells overproducing Wor1 (data not shown) with phospholipase activity being higher in MEA agar plates compared to SEA agar plates. In both media, *hog1* mutants show reduced phospholipase activity, with smaller hydrolysis halos compared to the wt strain that showed fivefold and 1.75-fold increase in size halos in both SEA and MEA agar plates, respectively. When *WOR1* is overexpressed, it results in an increase in the hydrolysis halos, behaviour independent of the background analysed (Figure 6b–c). No differences were found when incubation was done in microaerophilia (data not shown). Protease activity was analysed on BSA agar plates after 96 hours of incubation in normoxia and microaerophilia. Similarly, activity was lower in *hog1* mutants and overproduction of Wor1 increases protease production in both wt and *hog1* backgrounds (Figure 6a–d). BSA hydrolytic activity was slightly lower under microaerophilia for all the strains except for the *hog1* mutant. These data demonstrate that Wor1 overproduction renders cells with increased phospholipase and protease activities.

### *WOR1* overexpression does not restore virulence in *hog1* during a systemic infection

**Figure 7.**
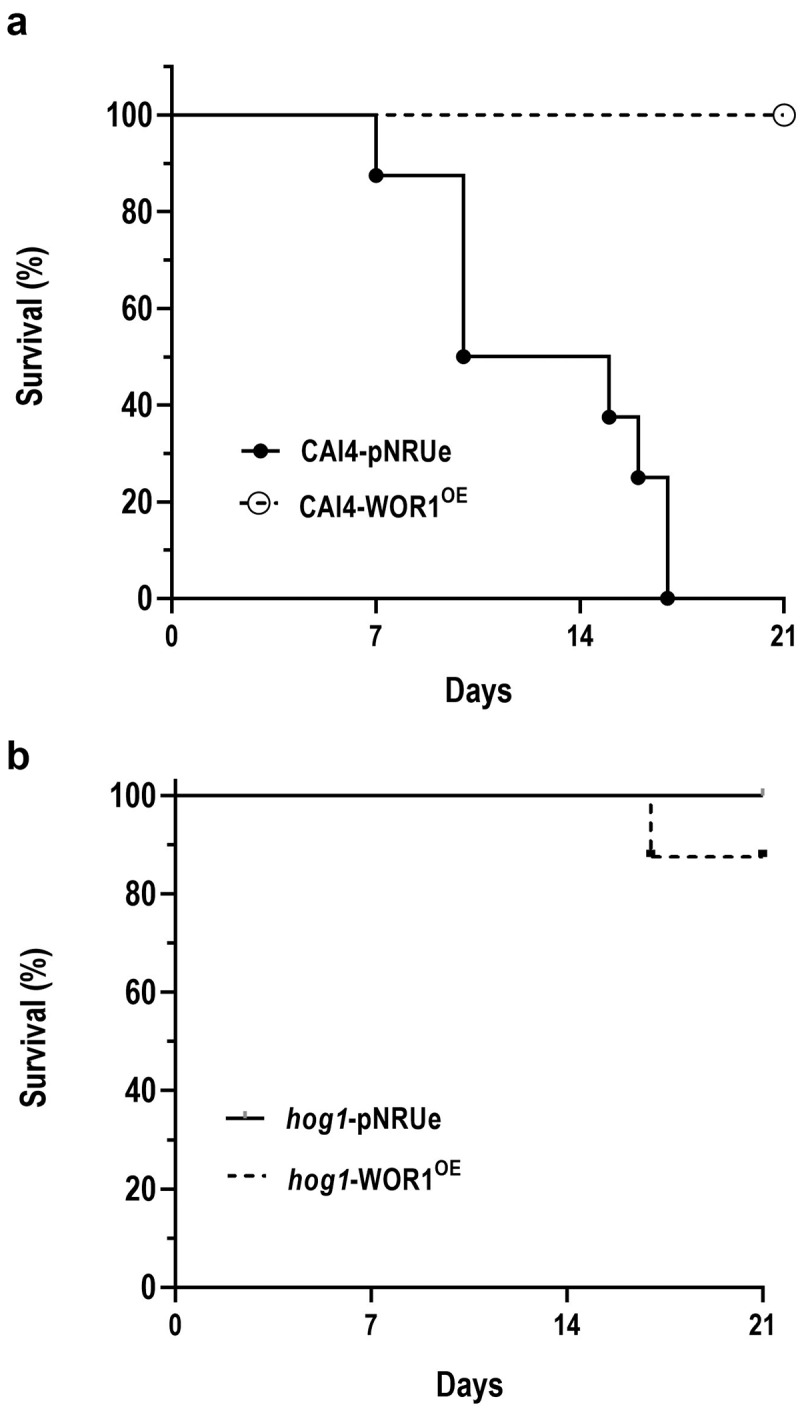
Effect of Wor1 overproduction in mouse viability (in a systemic candidiasis model).

Note: Survival curves of C57BL/6 systemically infected mice with 3x10^5^ cells of the indicated strains and data were plotted using the Kaplan–Meier method.

Since most of the invasive candidiasis have an endogenous origin, being the gut a potential portal of entry to the internal organs and *WOR1* overexpression results in increased fitness in this niche, we evaluated the role of WOR1^OE^ cells in a systemic candidiasis model. C57BL/6 mice were challenged with a lethal dose of CAI4-pNRUe, CAI4-*WOR1*^*OE*^, *hog1*-pNRUe, or *hog1*-WOR1^OE^ cells in the lateral vein of the tail. *WOR1* overexpression completely suppressed the CAI4 wild-type strain virulent phenotype (Figure 7a). It was previously reported that *hog1* showed a drastic increase in the mean survival time of infected mice with a ~ 60% of survival at day 60 after infection [37]. We confirmed those results as no mouse inoculated with *hog1* mutant cells died during the assay. *WOR1* overexpression did not have any effect in virulence, and death curves were like those obtained in *hog1* infected mice (Figure 7b). In summary, *WOR1* overexpression reduces the virulence in this model in wild-type cells, while it does not alter *hog1* virulence at the doses analysed.

## Discussion

The gastrointestinal tract is a complex environment with different physical and chemical stresses such as oxygen availability, pH changes, or detergents that commensal microorganisms must be able to cope with. In addition, this niche is subjected to fluctuations in the availability of nutrients, thus shaping a complex and diverse microbiota. As the HOG pathway is involved in sensing stress conditions both *in vitro* and *ex vivo*, it is perhaps not surprising to be crucial for *C. albicans* colonization of the mammalian gut [36]. We show here that *WOR1* overexpression restores the colonization capacity of this mutant, as *hog1* WOR1^OE^ cells can compete *hog1* and wt cells being able to permanently colonize the murine gastrointestinal tract of antibiotic treated mice (Figures 1 and 2). Which are the reasons for this behaviour?

We first paid attention to adhesion, a trait that is normally required for the persistence within a host of either commensals or pathogens. Adhesion of *C. albicans* to different surfaces is stronger upon hyphal induction. *hog1* mutants, although being hyperfilamentous, show reduced adhesion to the gut mucosa [36] and, as occurs in a wild-type background, Wor1 overproduction in this background increases adhesion to biotic (mice gut mucosa and epithelial cells) but not to abiotic (polystyrene) surfaces (Figure 3a) [29]. As *hog1* cells are also hyperfilamentous, this would suggest a specific expression pattern of cell surface proteins playing different roles in adhesion to cell lines and catheters. In fact, our qPCR data show that Wor1 overproduction causes a significant decrease in the expression of *ALS3* (in agreement with an *EFG1*-dependent expression) and other hyphae-related genes, such as *HWP1*, *ECE1,* and *EFG1*, but not *EAP1* or *ALS6*. Als3 is a hyphal specific adhesin and a virulence factor that binds specific receptors in host cells to induce endocytosis [42] and is repressed in *hog1* cells. However, we show here that it is not essential for the cells to adhere to the intestinal mucosa and it does not alter fungal loads in the mouse gut (Figure 4) in agreement to recent data [38]. *HWP1* encodes a hyphal protein that mediates attachment to oral epithelial cells via host transglutaminase activity [43]; Hwp1 is not involved in virulence in an animal model, but it is partially defective in translocation across the intestinal tract [44]. Among ALS proteins, Als1, which is not limited to a specific morphological form, and the hyphal specific Als3 seem to be the most important proteins involved in adhesion to epithelial cells as *C. albicans* knockout mutants adhere less efficiently and their overexpression in *S. cerevisiae* influence adhesion [45, 46]. Our work suggests that the high expression *ALS3* could be detrimental for the establishment of *C. albicans* as a commensal. Reduced expression of *ALS3* and *ALS1* adhesins is in line with recent evidence indicating that mucosal IgA are crucial in controlling *C. albicans* colonization in the gut by targeting hyphae-specific epitopes [38, 47]. However, caution must be taken between *in vitro* and *in vivo* analyses of protein abundance. For example, mucins have inhibitory effects on adherence to biotic surfaces [39] and could influence *Candida* spp. colonization. Wor1 overproduction increases adherence to both the mouse intestine and the HT29 cell line (Figure 4), which does not produce mucus and this effect is higher in our mouse *ex vivo* tissue model. Secretory aspartyl protease Sap2, shown to counterbalance the effect of mucins in the interaction of *C. albicans* with human buccal epithelial cells [48], could also play a role in mammalian gastrointestinal colonization, especially as protease activities are augmented in *WOR1*^OE^ cells.

A second important aspect regulating pathogenesis and commensalism is dimorphism. Filamentation seems to be dispensable for murine gut colonization [49–52] and long-term gastrointestinal colonization of *C. albicans* cells favours the selection of deficient-filamentous fungi [40]. According to this, we show here that the increased fitness of *hog1* WOR1^OE^ cells could be due to the repression of the hyperfilamentous phenotype of *hog1*. A connection between mutations affecting *FLO8* and decreased expression of *SAP6* has been recently postulated to be important for enhanced fitness phenotypes of evolved *C. albicans* cells upon serial passage within the murine gut [40, 49]; however, the role of Wor1 and Hog1 in this network remains unknown. The dominant repressor role of Hog1 has been previously described to rely on elements other than those regulated by *EFG1*, and *efg1 hog1* double mutants are still hyperfilamentous [34]. In any case, overexpression of *WOR1* in *efg1 hog1* mutants also inhibits filamentation in vitro and partially recovers colonization in the commensalism model (data not shown), suggesting that defects in dimorphism *in vitro* do not necessarily imply a defect *in vivo* and different groups have recently supported this idea [49, 53–55]. Nevertheless, it has been shown that when inducing filamentation *in vivo* by repressing *TUP1* using a *TUP1*-regulated strain, cells are completely lost in the mice gut, and therefore, it suggests that the yeast form has advantages over the hyphal form in the ability to colonize [52].

Metabolic adaptation may also be invoked to explain, at least partially, our results. Although glucose is a preferred carbon source for many fungi, this sugar is limited in the gut and fungi reorientate their metabolism towards alternative carbon sources. Metabolism has been shown to play a role in *WOR1*-mediated adaptation [19] and overproduction of Wor1 results in an alternative respiratory metabolism with a decrease in the use of certain fermentable carbon sources by downregulation of the glyoxylate cycle [30]. The use of alternative non-fermentable carbon sources, such as lactate, N-acetylglucosamine (a component of host mucin), amino acids, or organic acids produced by microbial fermentation requires a functional mitochondrial complex I to coordinate assimilation pathways. WOR1^OE^ cells, as *hog1* mutants, are more sensitive to electron-chain inhibitors [29, 56] and *hog1* cells show an altered metabolism that results in higher ROS levels and dependence on mitochondrial ATP synthesis under growth *in vitro* [56]. Similarly, *WOR1*^OE^ causes defects in oxidative metabolism and defect in alternative carbon usage. How these metabolic alterations promote enhanced survival in the murine gut remains unknown.

We finally present that *WOR1*^OE^ also reduces wild-type virulence during a systemic infection in a wild type and retains the attenuated virulence of *hog1* mutants in this experimental model, which has been related by defects in the oxidative stress response that *hog1* cells display [37, 57]. According to that, *WOR1* overexpression in both wild type and *hog1* renders in an increased susceptibility to oxidants (data not shown).

Based on our results, it is an open question whether Hog1 and Wor1 are interconnected in some way. Liang *et al*. demonstrated that deletion of *HOG1* in *MTL* homozygous **a**/**a** or *α*/*α* cells promotes conversion to the opaque state by mechanisms dependent on Wor1 [58], clearly establishing a link between both genes. However, caution must be taken while interpreting this connection, since GUT cells differ from opaque cells (morphologically and transcriptionally) and they are not phenotypically equivalent to the opaque cells studied in Liang et al. [58]. In vitro transcriptional analysis in *hog1* mutants revealed no differences in *WOR1* expression compared to a wild-type strain under different stress conditions [59]. An alternative explanation is that the improved colonization in *hog1* mutants caused by *WOR1* overexpression is due to secondary effects. The consequences of altering these genes are very diverse, including changes in the cell wall and membranes, and altered morphogenesis and metabolism (among others), all of which can be clearly related to the colonization of this niche.

In conclusion, we present here evidence that *WOR1*^OE^ in *C. albicans* results in a profound reorientation of the fungal cell towards commensalism by acting on several pathogenicity factors. Given the potential benefits of *C. albicans* colonization [8] in human health, this opens the possibility of using avirulent commensals in our benefit and/or for therapeutical interventions upon gut colonization.

## Materials and methods

### Strains and growth conditions

The strains used are described in Table 1. *Candida* cells were grown at 37ºC in either liquid or solid media in YPD (1% yeast extract, 2% peptone, and 2% dextrose). SD-chloramphenicol (2% dextrose, 0.5% ammonium sulphate, 0.17% yeast nitrogen base supplemented with amino acids, 2% agar, and 20 µg/mL chloramphenicol) plates were used for CFUs counting. The susceptibility/resistance to different compounds was performed by standard drop test as follows. Stationary or exponential phase (O.D. = 1) growing cells were adjusted to 2 × 10^7^ cells/mL, and 5 µL of tenfold serial dilutions were deposited onto solid YPD plates supplemented (or not) with the indicated compounds. Plates were incubated at 37ºC for 24 and 48 hours before being scanned. For the observation of white or opaque phenotypes, *C. albicans* strains were grown in YPD plates supplemented with phloxine B (10 µg/L) with or without doxycycline (10 µg/ml) at 37 ºC. When necessary, doxycycline was added to liquid at 10 µg/L. To determine the phospholipase and protease activity, ~ 5 × 10^4^ cells from O/N grown cultures were deposited on the MEA (Malt extract agar) 6.5%; egg-yolk 2%; NaCl 1–0.5 M; peptone 0.1%; dextrose 2%; CaCl_2_ 0.055%) [63], SEA (Sabouraud chloramphenicol agar) 6.5%; egg-yolk 2%; NaCl 1–0.5 M; CaCl_2_ 0.0055%) [63], or BSA (yeast carbon base 1.17%; YNB 0.01%; agar 2%) agar plates and incubated for 96 hours (MEA, SEA) or 120 hours (BSA) at 37°C under normoxia or microaerophilia atmospheres before halos were measured. The plates were further scanned. Microaerophilia environment was achieved using a commercial system in an anaerobic chamber (GENBox Microaer). Yeast-to-hypha transition *in vitro* was induced either from cells growing at 30ºC and inducing filamentation with low inoculum in YPD media at 37ºC or by growing cells in YPD plus foetal bovine serum at 37ºC. Samples were collected at different time points, fixed with 4% formaldehyde before being photographed under optical microscopy

**Table 1.** *Candida albicans* strains used in this work.

Strain	Genotype	Reference
CAI4	*ura3:imm434/ura3:imm434 iro1/iro1:imm434*	[60]
CAI4-RFP	[CAI4] *ADH1/adh1:TDH3*^*PR*^*tTA TET*^*PR*^*-dTOM2-URA3*	[29]
CAI4-pNRUe (REP40)	[CAI4] *ADH1/adh1:tTA-TET*^*PR*^*-URA3*	[61]
CAI4- WOR1^OE^	[CAI4] *ADH1/adh1:TDH3*^*PR*^*tTA TET*^*PR*^*-WOR1-myc-URA3*	[29]
HI7	[CAI4] *hog1:hisG/hog1:hisG*	[36]
*hog1*-RFP	[HI7] *ADH1/adh1:TDH3*^*PR*^*tTA TET*^*PR*^*-dTOM2- URA3*	This study
*hog1-*pNRUe	[HI7] *ADH1/adh1:tTA-TET*^*PR*^*-URA3*	This study
*hog1*-WOR1^OE^	[HI7] *ADH1/adh1:TDH3*^*PR*^*tTA TET*^*PR*^*-WOR1-myc-URA3*	This study
*als3* (1954)	[CAI4] *als3sa*Δ/*als3sa*Δ-*URA3*	[62]

### Genetic procedures

A *hog1* mutant overexpressing *WOR1* (*hog1*-WOR1^OE^) was obtained by integrating at the *ADH1* region a *Kpn* I-*Sac* II fragment of pNRUX-WOR1 plasmid [29]. Two independent *WOR1*^*OE*^ clones were generated with similar expression levels and *in vitro* phenotypes but only one (clone 1) was used for animal infection studies.

### Protein extracts and immunoblot analysis

Cell lysis, protein extraction, separation in SDS-PAGE, and transfer to nitrocellulose membranes were performed as described [64, 65]. Protein quantification was achieved by measuring the absorbance at A_280 nm_ and equal amount of proteins were loaded for immunoblots with anti-myc, clone 4A6 (Millipore). Western blots were developed according to the manufacturer’s conditions using the Hybond ECL kit (Amersham Pharmacia Biotech).

### Quantitative PCR (qPCR)

Quantitative reverse transcription-PCR assay was performed following the protocol described previously [60, 66]. Primer Express Software 2.0 (Applied Biosystems) was used to select pair of primers for specific amplification of the internal control *ACT1* [act1-for (RT): 5´-TGGTGGTTCTATCTTGGCTTCA; act1-rev (RT): 5´-ATCCACATTTGTTGGAAAGTAGA], *WOR1* [wor1-for (RT): 5´-CAAATATGGCAGTGAATTCAAGTT; wor1-rev (RT): 5´- TGGCATGGGTTCATATTCG]; *EFG1* [efg1-for (RT): 5´- ACAACCAACAACAACAGGCA; efg1-rev (RT): 5´- GCAAACAACTGCAGCCAAT], *ALS3* [als3-for (RT): 5´- CTAATGCTGCTACGTATAATT; als3-rev (RT): 5´- CCTGAAATTGACATGTAGCA], *ALS6* [als6-for (RT): 5´- TTCGGATACCAGCATTAGCTCA; als6-rev (RT): 5´- CGACCCAGCATTAATATTGCC], *EAP1* [eap1-for (RT): 5´- CTGCTCACTCAACTTCAATTGTCG; eap1-rev (RT): 5´- GAACACATCCACCTTCGGGA], *ECE1* [ece1-for(RT): 5´- TCAGCTGAATCTGCTTTGAAAGA; ece1-rev (RT): 5´- GTGCTACTGAGCCGGCATC].

### In vivo procedures

In all, 7–10 weeks old female mice C57BL/6 from Harlan Laboratories, Inc. (Italy), were housed in the animal facility of the Medical School of the Universidad Complutense de Madrid. Animal procedures were performed in accordance with the “Real Decreto 1201/2005, BOE 252” for the Care and Use of Laboratory Animals of the “Ministerio de la Presidencia,” Spain, and protocols used were approved by the Animal Experimentation Committee of the University Complutense of Madrid and Comunidad de Madrid according to Artículo 34 del RD 53/2013 (PROEX 226/15 and PROEX120/19). We used the minimal number of animals per experiment to obtain results with statistical validity and procedures experimental procedures were carried out minimizing mice suffering.

Gastrointestinal colonization and relative proportions of strains in stomach, small and large intestines were performed as previously described [36]. The relative colonization ratio of strain A versus the competing strain B in a specific X region (stomach, proximal small intestine, distal small intestine, caecum, and large intestine), RCR^A^_X_, was calculated as follows: (Fungal load strain A/Fungal load strain B) at location X/(Fungal load strain A/Fungal load strain B) at large intestine. For virulence assays, we used the standard mice systemic infectious model [61,62]. Cells were obtained from overnight cultures grown in YPD at 37°C, washed twice with PBS, and 250 μL containing 3 × 10^5^ CFUs were inoculated into the lateral tail vein of the mice. Animals were monitored daily and killed by CO_2_ suffocation upon clear signs of disease (laziness, disorientation, weight loss) following standard protocols (AVMA Guidelines for the Euthanasia of Animals: 2013 Edition). A Kaplan – Meier survival analysis was used to estimate virulence.

### Adhesion assays

For adhesion to either polystyrene or intestinal mucosa we followed protocols described previously [29,36]. Human colon adenocarcinoma cell-line HT29 was grown on a DMEM medium with 25 mM glucose (Gibco, Waltham, MA, USA), 10% heat-inactivated foetal bovine serum, 100 units/mL penicillin, and 100 µg/mL streptomycin in a humidified incubator with 5% CO_2_ atmosphere at 37ºC. Once confluency was achieved, culture medium was removed, and 1 mL/well of the same medium without serum was added. Overnight grown *C. albicans* cells were washed twice with PBS, counted, and resuspended in DMEM medium lacking serum. An equal amount of RFP labelled CAF2 or *hog1* and CAF2-WOR1 or *hog1*-WOR1 was prepared, and 2 × 10^5^ cells/well were added. After 1 hour of incubation at 37ºC in a humidified incubator with 5% CO_2_ atmosphere, culture medium was removed, and cells attached to the HT29 cell line were mechanically removed by adding 500 µL of water with 0.2% Triton twice. In all, 100 µL from different dilutions were plated on SD agar plated, and CFUs were counted. Adhesion is expressed by the Adherence Relative Index (ARI) obtained by dividing the percentage of adhered cells of each strain by their percentage in the inoculum.

### Statistical analysis

Statistical differences between two groups for colonization fitness studies were calculated using Student’s two-tailed unpaired t-tests, with a type I error value (α) = 0.05. Computations assume that all rows are sampled from populations with the same scatter (SD). When comparing more than two groups, ordinary one-way ANOVA was used for multiple comparisons using the method with α = 0.05.

## Supplementary Material

Supplemental Material

## Data Availability

The authors confirm that the data supporting the findings of this study are available within the article [and/or] its supplementary materials.
