# Supplementary figures and images for: The defective gut colonization of Candida albicans hog1 MAPK mutants is restored by overexpressing the transcriptional regulator of the white opaque transition WOR1
Source: Virulence. 2023 Feb 9;14(1):2174294. doi: 10.1080/21505594.2023.2174294 (PMC9928469; doi:10.1080/21505594.2023.2174294)

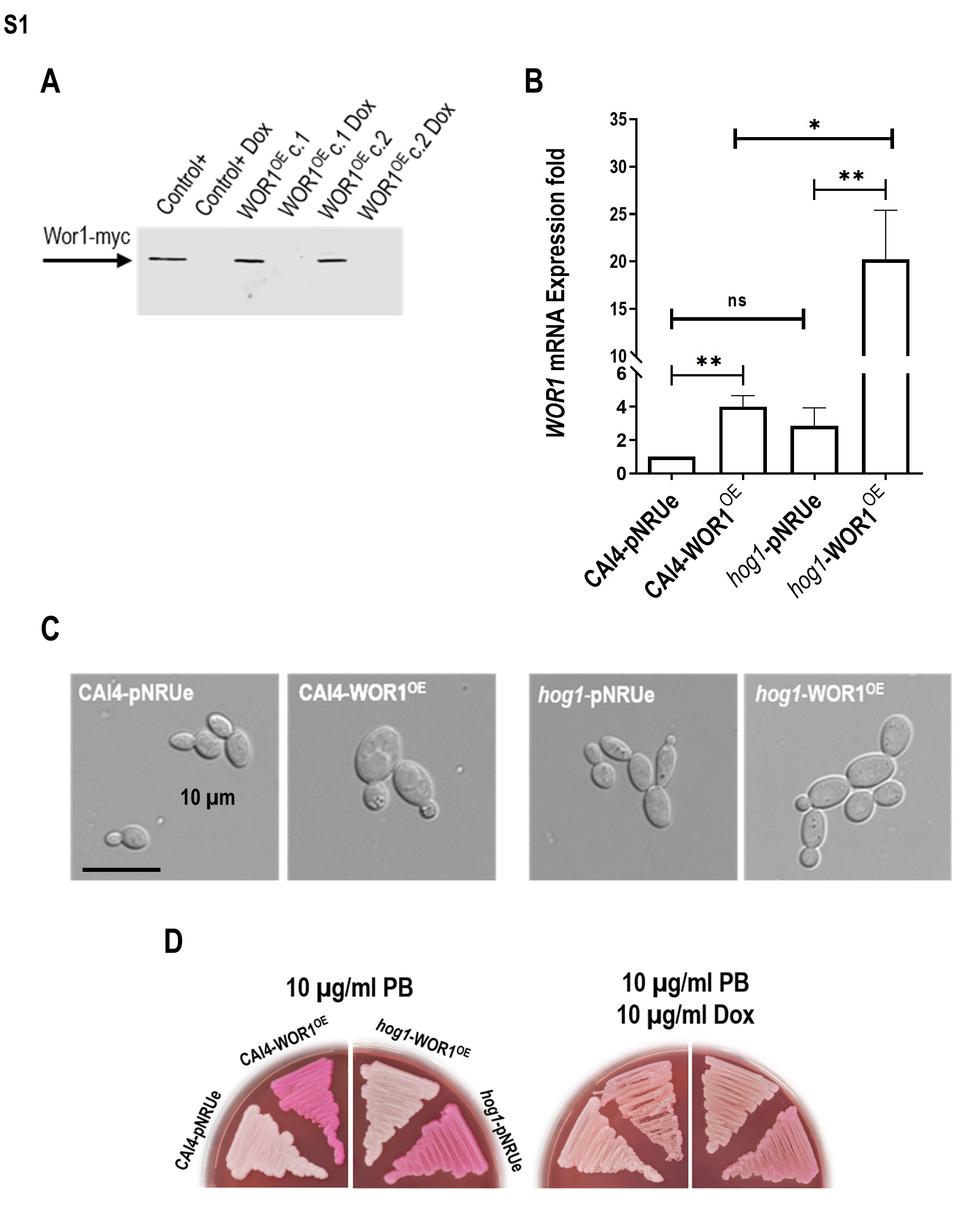

Supplement: Supplemental Material [file KVIR_A_2174294_SM5470.zip › Figure S1.jpg]

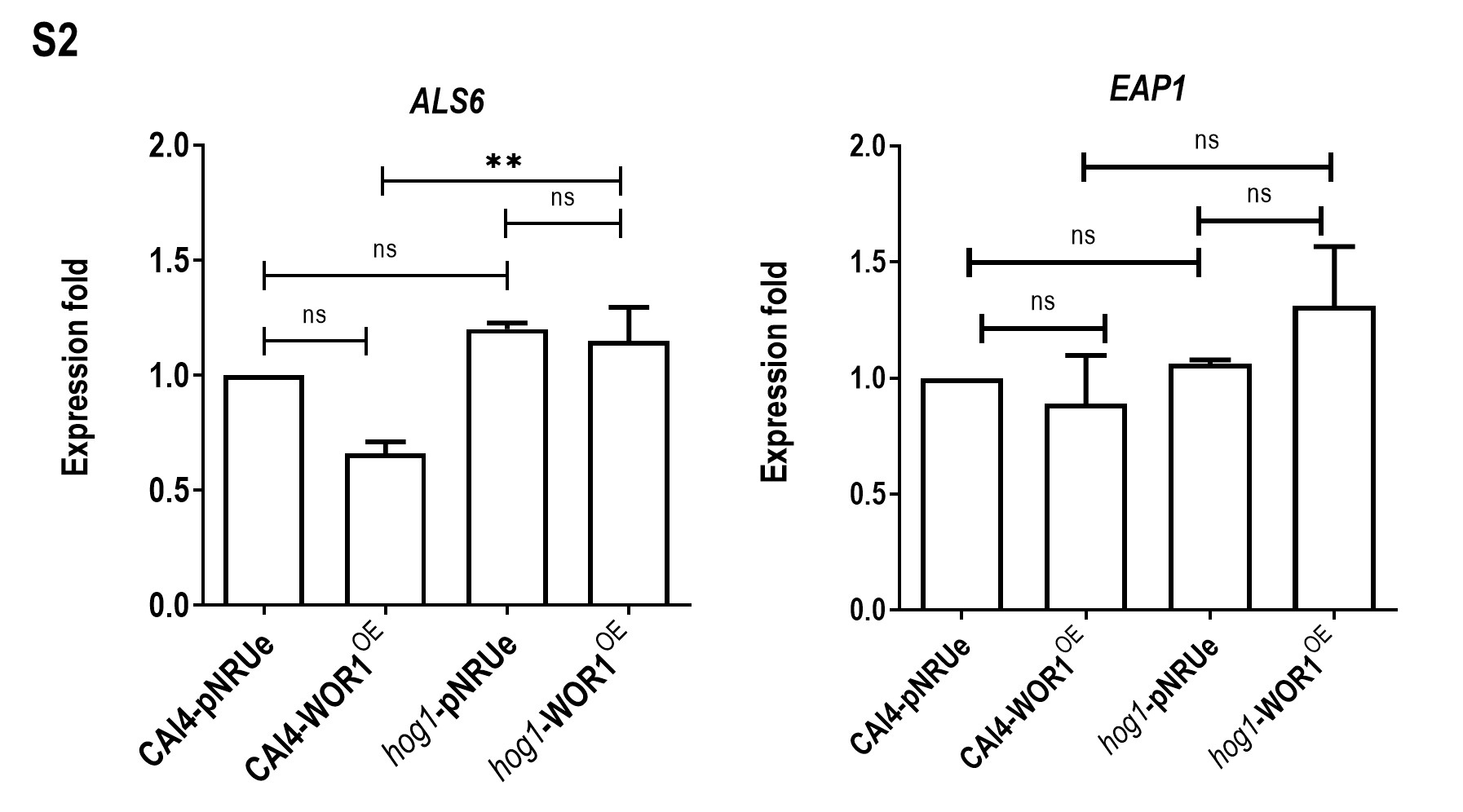

Supplement: Supplemental Material [file KVIR_A_2174294_SM5470.zip › Figure S2.jpg]

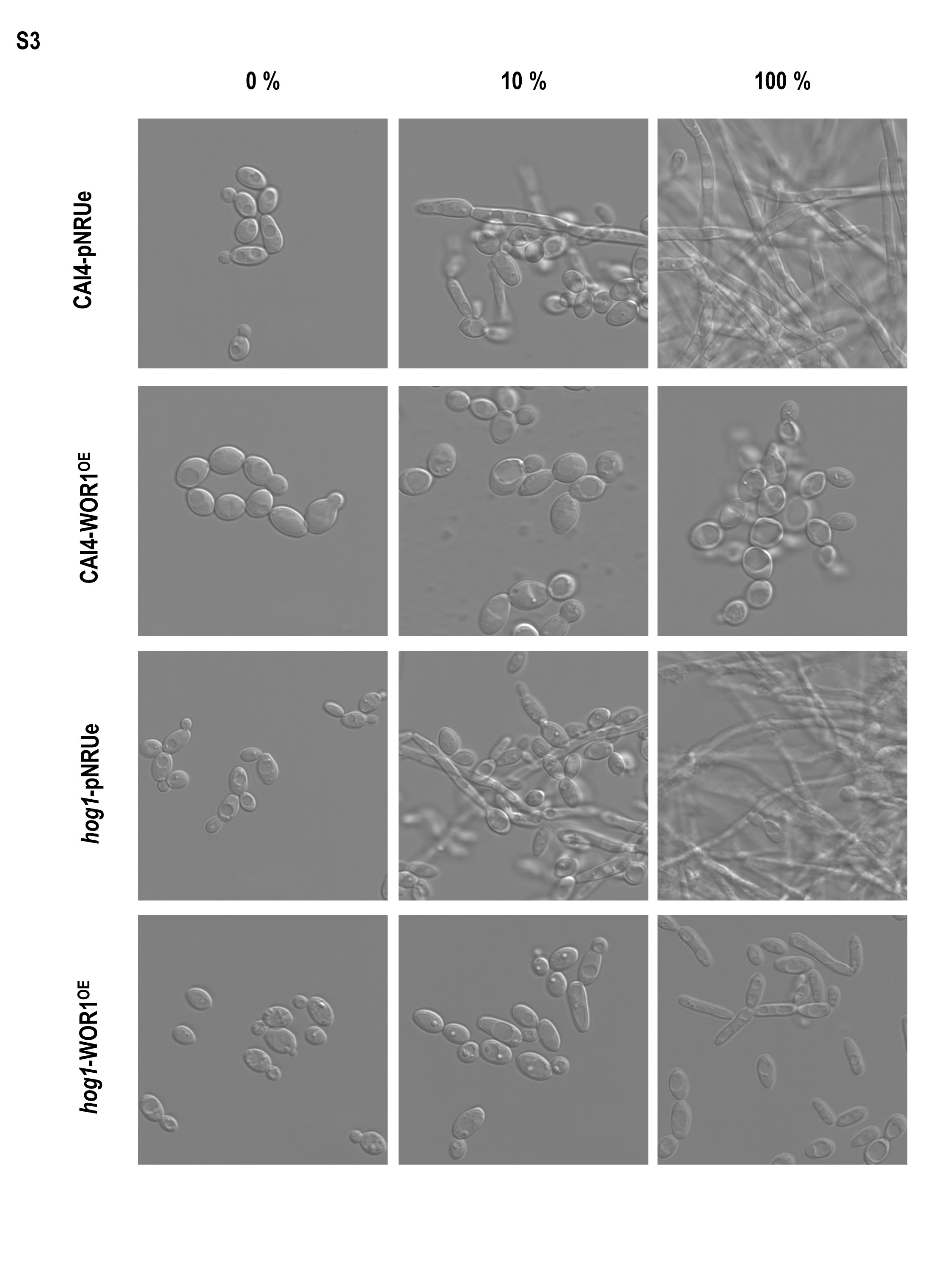

Supplement: Supplemental Material [file KVIR_A_2174294_SM5470.zip › Figure S3.JPG]
